# Intravitreal cilium associated with retinal detachment 40 years following penetrating eye injury: a case report

**DOI:** 10.1186/s12886-015-0010-6

**Published:** 2015-03-12

**Authors:** Maria Dettoraki, Konstantinos Andreanos, Stavroula Davou, Nikolaos Nomikarios, Marilita M Moschos, Dimitrios Brouzas

**Affiliations:** 1st Department of Ophthalmology, University of Athens, “G. Gennimatas” General Hospital of Athens, 154 Mesogion Av, 11527 Athens, Greece

**Keywords:** Cilium, Vitreous cavity, Retinal detachment, Penetrating eye injury

## Abstract

**Background:**

The presence of an intraocular cilium is very rare and the response of the eye to the cilium is variable. We present the case of a patient with a cilium found in the vitreous cavity during vitrectomy for rhegmatogenous retinal detachment 40 years following penetrating eye injury. To our knowledge, this is the longest reported presence of a cilium in the vitreous cavity.

**Case presentation:**

A 70-year-old Caucasian woman presented to the emergency department of our hospital complaining of sudden visual impairment and floaters of her right eye initiated 2 weeks earlier. Ophthalmic history included a penetrating injury of the right eye with a sharp metallic object 40 years ago and an uncomplicated phacoemulsification surgery in the same eye 2 years earlier. Fundoscopy revealed an inferior macula off rhegmatogenous retinal detachment. No inflammation was present. During vitrectomy and under scleral indentation at 5-o’clock position, a cilium was found at far retinal periphery. One end of the cilium was embedded in the retina, whereas the other end floated freely in the vitreous. The cilium was removed through the pars plana sclerotomy with intraocular foreign body forceps. The procedure was completed without any complications.

**Conclusion:**

Penetrating eye injury is the most possible cause of cilium entrance in vitreous cavity in this case, which suggests that cilium can be well tolerated in vitreous cavity for as long as 40 years.

## Background

The presence of cilia intraocularly has been rarely associated with penetrating eye injuries. Retained cilia have been reported in the anterior [1] and posterior chambers [2], iris [3], lens [4], vitreous cavity [5] and retina [6,7]. Moreover, intraocular cilia have been reported without apparent etiology, since no history of trauma or eye surgery existed [5,8]. The response of the eye to the eyelash foreign body is variable. Cases of acute or delayed inflammatory responses [9], endophthalmitis [8,10] and bullous keratopathy [3] have been reported, whereas cases of cilia remaining dormant in the eye for long periods have also been described [11-13]. Up to now, the longest reported presence of an eyelash in the vitreous cavity without eliciting any response is 32 years [3].

We present the case of a cilium found in the vitreous cavity of a 70 year old Caucasian female patient. We believe that the cilium in question entered in the vitreous cavity as a result of penetrating injury 40 years prior to the time of the vitrectomy. Other less likely ways of entry include phacoemulsification performed 2 years prior to beginning of symptoms or even during the pars plana vitrectomy.

## Case presentation

A 70-year-old Caucasian woman presented to the emergency department of our hospital complaining of sudden visual impairment and floaters of her right eye initiated 2 weeks earlier. Ophthalmic history included a penetrating injury of the right eye with a sharp metallic object 40 years ago and an uncomplicated phacoemulsification surgery in the same eye 2 years earlier. The left eye was normal and the medical history was unremarkable.

**Figure 1:**
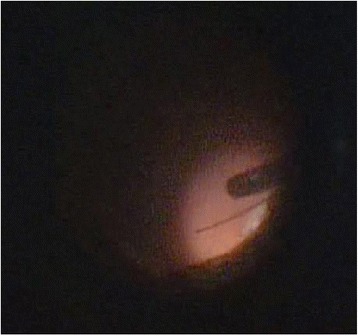
**Cilium in the vitreous cavity.** During vitrectomy a cilium was found in the vitreous cavity with one end embedded in the retina and the other end floated in vitreous.

**Figure 2:**
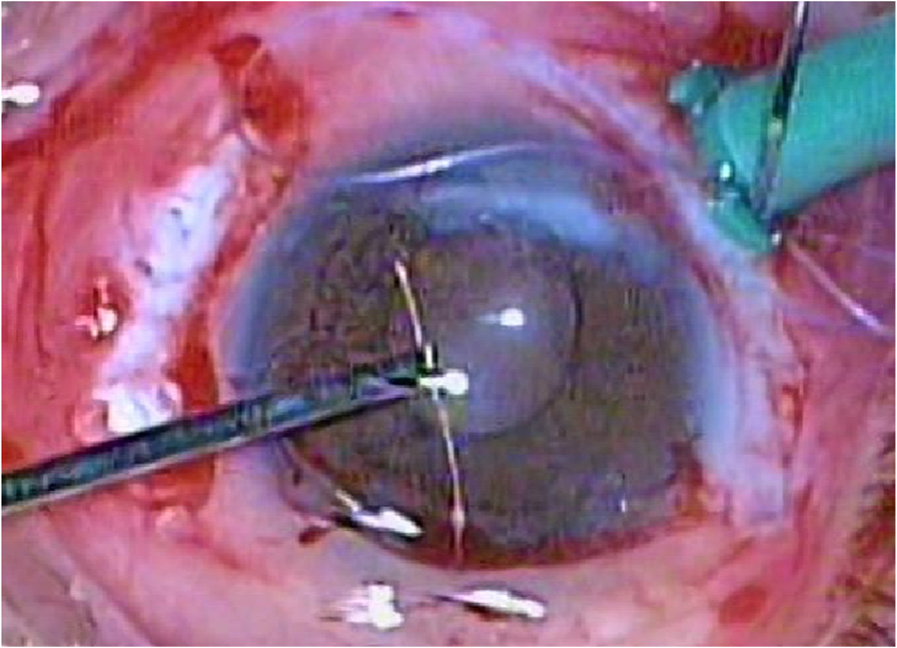
**Removal of cilium with intraocular foreign body forceps.** The cilium was removed through the pars plana sclerotomy with intraocular foreign body forceps.

**Figure 3:**
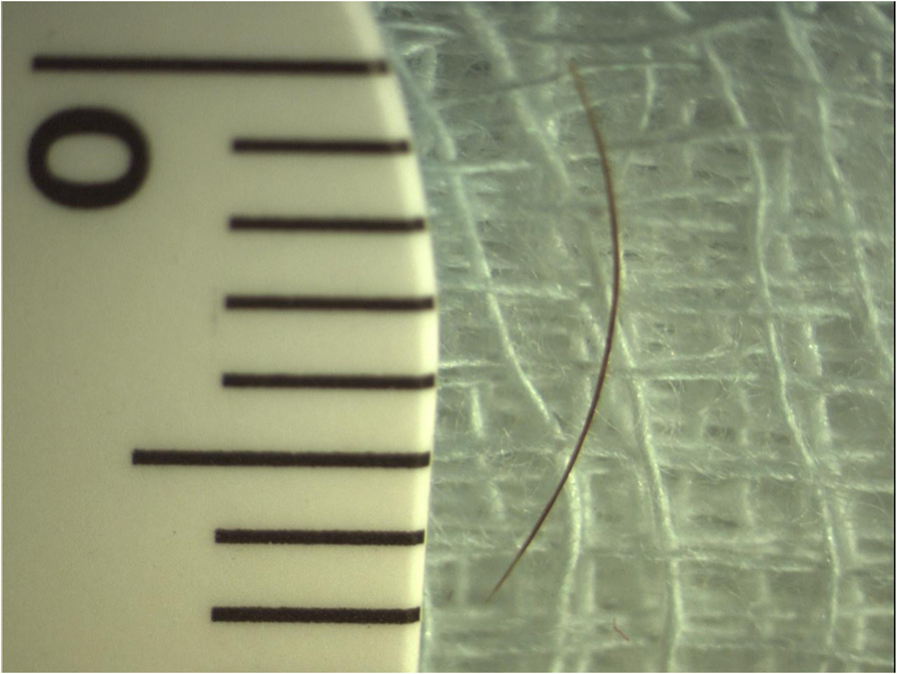
**The 7 mm-length cilium removed from the vitreous cavity.** The cilium appeared intact 40 years after its entrance in the vitreous cavity.

At presentation, visual acuity was “hand motion” in the right eye and slit-lamp examination revealed an opacity at the inferior ¼ of the cornea due to past injury and a posterior chamber intraocular lens with an intact posterior capsule. Intraocular pressure was 12 mmHg. Fundoscopy revealed an inferior macula off rhegmatogenous retinal detachment. There was no evidence of intraocular inflammation. Two days later, the patient underwent 20G three-port pars plana vitrectomy in the right eye under retrobulbar anaesthesia. During vitrectomy and under scleral indentation at 5-o’clock position, a cilium was found at far retinal periphery. One end of the cilium was embedded in the retina, whereas the other end floated freely in the vitreous (Figure 1). The cilium was cleared of the surrounding vitreous with the vitreotome and was carefully removed through the pars plana sclerotomy with intraocular foreign body forceps (Figure 2). The 7 mm-length cilium was completely intact, retaining its shape and colour (Figure 3). The procedure was completed without any complications.

## Discussion

We present the case of a patient with a cilium in the vitreous cavity and partly embedded in the retina discovered during pars plana vitrectomy for rhegmatogenous retinal detachment.

Previous reports have described the presence of intraocular cilia secondary to penetrating trauma [3,9,14], surgical intervention [1,2,10] or even without apparent etiology [5,8]. Gupta et al. [6] described a case of 2 cilia embedded in the retina 1 month after the repair of an ocular perforation. Similarly, Humayun et al. [3] presented 5 cases with intraocular cilia as a result of penetrating injury. In one of these cases, the cilium was noted in the anterior vitreous 32 years after the trauma, which is the longest reported presence of an intravitreal cilium up to now.

Phacoemulsification is a possible way of foreign body introduction into the eye. Islam and Dabbagh [1] observed a cilium within the anterior chamber of a patient 3 months after phacoemulsification surgery and implantation of a posterior chamber intraocular lens. Rofail et al. [12], described a migratory intraocular cilium 3 days following phacoemulsification. It was initially noted in the anterior chamber, subsequently pierced the iris, migrated to the posterior chamber and finally re-emerged through the pupil and rested in the inferior angle. The authors proposed postoperative eye rubbing as the mechanism of cilium entry through the cataract incision.

In our case, the eyelash was found under scleral indentation at the far inferior retinal periphery. Moreover, an opacification was present in the inferior ¼ of the cornea adjacent to the area where the eyelash was detected. This is in favor of the hypothesis that the eyelash was embedded at the time of penetrating eye injury 40 years ago.

Our patient also underwent an uncomplicated phacoemulsification surgery 2 years earlier. In reported cases of intraocular cilium related to phacoemulsification surgery, its location was limited to the anterior segment of the eye and most commonly in the anterior chamber [1,3,12]. Therefore, in our case, it is unlikely that the cilium entered the eye through the cataract incision, since it was located at the far retinal periphery with one end fixed firmly in the retina.

The prognosis of intraocular cilia is variable. It ranges from lack of any response to acute inflammation [9], endophthalmitis [8] and retinal detachment [15]. In our case, rhegmatogenous retinal detachment occurred. However, we cannot propose that the cilium was directly related to such complication, since the past history of the patient included a penetrating trauma and two surgical interventions, the trauma repair and the phacoemulsification surgery, all of which predispose to retinal detachment.

## Conclusion

We conclude that penetrating eye injury is the most possible cause of cilium entrance in vitreous cavity in this case, which suggests that cilium can be well tolerated in vitreous cavity for as long as 40 years.

## Consent

Written informed consent was obtained from the patient for publication of this case report and any accompanying images. A copy of the written consent is available for review by the Editor of this journal.
